# Community Health Worker-Led mHealth-Enabled Diabetes Self-management Education and Support Intervention in Rural Latino Adults: Single-Arm Feasibility Trial

**DOI:** 10.2196/37534

**Published:** 2022-05-30

**Authors:** Shiyu Li, Zenong Yin, Janna Lesser, Chengdong Li, Byeong Yeob Choi, Deborah Parra-Medina, Belinda Flores, Brittany Dennis, Jing Wang

**Affiliations:** 1 Center on Smart and Connected Health Technologies School of Nursing University of Texas Health Science Center at San Antonio San Antonio, TX United States; 2 Department of Public Health The University of Texas at San Antonio San Antonio, TX United States; 3 School of Nursing University of Texas Health Science Center at San Antonio San Antonio, TX United States; 4 College of Nursing Florida State University Tallahassee, FL United States; 5 Department of Population Health Sciences University of Texas Health Science Center at San Antonio San Antonio, TX United States; 6 Latino Research Institute The University of Texas at Austin Austin, TX United States; 7 South Coastal Area Health Education Center Corpus Christi, TX United States

**Keywords:** health disparity, rural health, rural, community health worker, health education, digital health, diabetes, diabetes management, mHealth, community health, self management, mobile health, technology feasibility, underserved, Latino

## Abstract

**Background:**

Latinos living in rural South Texas have a higher prevalence of diabetes, but their access to diabetes self-management education and support (DSMES) is limited.

**Objective:**

We aimed to test the feasibility of a community health worker-led, mobile health (mHealth)-based DSMES intervention to reduce disparities in accessing DSMES in underserved rural Latino residents in South Texas.

**Methods:**

This 12-week, single-arm, pre-post trial was delivered by trained community health workers to 15 adults with type 2 diabetes. The intervention consisted of digital diabetes education, self-monitoring, a cloud-based connected platform, and community health worker support. Feasibility was evaluated as retention, actual intervention use, program satisfaction, and barriers to implementation. We also explored the intervention’s effect on weight loss and hemoglobin A_1c_ (HbA_1c_).

**Results:**

All 15 participants were Latino (mean age 61.87 years, SD 10.67; 9/15 female, 60%). The retention rate at posttest was 14 of 15 (93%). On average, the participants completed 37 of 42 (88%) digital diabetes education lessons with 8 participants completing all lessons. Participants spent 81/91 days (89%) step tracking, 71/91 days (78%) food logging, 43/91 days (47%) blood glucose self-monitoring, and 74/91 days (81%) weight self-monitoring. The level of program satisfaction was high. On average, participants lost 3.5 (SD 3.2) kg of body weight (*P*=.001), while HbA_1c_ level remained unchanged from baseline (6.91%, SD 1.28%) to posttest (7.04%, SD 1.66%; *P*=.668).

**Conclusions:**

A community health worker-led mHealth-based intervention was feasible and acceptable to improve access to DSMES services for Latino adults living in rural communities. Future randomized controlled trials are needed to test intervention efficacy on weight loss and glycemic control.

## Introduction

Diabetes is a complex and costly disease that requires persons with diabetes to make daily self-care decisions to prevent the onset of complications [1]. Diabetes self-management education and support (DSMES) is the ongoing process of offering knowledge, skills, and support for diabetes self-care. Improving access to DSMES could empower persons with diabetes to self-manage diabetes and improve their health [2]. This is particularly important for residents of South Texas, who have a higher prevalence of diabetes than the rest of Texas (11.6% vs 9.3%) or the United States overall (8.9%) [3].

South Texas has 38 counties, of which 25 are rural. Diabetes care disparities exist in rural South Texas for a variety of reasons. Rural persons with diabetes frequently lack adequate transportation and must travel long distances to clinics, impeding diabetes care and potentially impacting glycemic control [4,5]. Meanwhile, rural residents are poorer, making active participation in routine care more difficult [4,6]; empirical evidence suggests that rural patients are more likely to defer care due to limited financial resources than their urban counterparts [7]. Furthermore, Texas has a significant physician shortage in rural areas which has been exacerbated by the COVID-19 pandemic [8]. Rural residents rely heavily on federally funded health care programs, resulting in much lower reimbursement payments for rural physicians and hospitals than their urban counterparts and a diminished desire to work in rural areas [4]. Additionally, rural residents’ low levels of education and literacy may hinder their capacity to comprehend diabetes self-management knowledge and skills [6]. An aging population may also exacerbate these barriers in rural Texas; many elderly residents have decreased cognitive function and suffer from diabetes comorbidities [9,10].

More than 80% of the South Texas population is Latino. Latinos face additional cultural barriers when it comes to diabetes care [11]. The lack of cultural competence among health care providers has been extensively documented as a barrier for Latinos during clinical encounters, potentially contributing to lower patient satisfaction and disengagement from care [11,12]. A lack of linguistic proficiency has been linked to inadequate diabetes care for Latinos in several studies [13]. For example, Lopez-Quintero et al [12] found that non–Spanish speaking providers are less likely than Spanish-speaking physicians to provide physical activity and diet recommendations to their Latino patients. Furthermore, switching to a healthy diet from their traditional Latino cuisine is difficult for Latinos with type 2 diabetes [14]. Rice, beans, and tortillas, which are high in refined carbohydrates, are staples of traditional Latino food [15]. A qualitative study done by Hu et al [14] reported that one of the most significant hurdles to healthy diet adherence is overcoming cravings for traditional foods. Diet adherence is also impeded by the importance of family support in Latino culture, particularly for women, who have been reported to experience family conflicts about dietary issues, such as keeping their husband and children happy while adhering to diet restrictions [11,14,15].

Improving access to DSMES for rural Latino population requires applying a multidimensional research lens. The National Institute on Minority Health and Health Disparities framework conceptualizes multiple domains of health determinants that affect minority health [16]. According to the framework, to address access barriers for the rural Latino population, equity-oriented strategies are necessary that may include addressing individual beliefs and attitudes pertaining to diabetes education–seeking behavior (behavioral-individual), familial norms about diet and exercise (behavioral-interpersonal), Latino culture–specific norms that hinder diabetes self-management (sociocultural environment-community), limited language proficiency and literacy level (sociocultural environment-individual), uninsured status (health care system-individual), limited access to DSMES services (health care system-community), and state and federal policies pertaining to local communities (behavioral-societal and health care system-societal) [16].

Community health worker-led DSMES is a culturally-appropriate and cost-effective approach for improving access to DSMES services among rural and minority populations [17]. Community health workers are community members trained to provide culturally appropriate health education. As trusted community members sharing similar cultural and linguistic backgrounds with persons with diabetes, community health workers can provide individual-level emotional support and instrumental support [18]. They also have a unique ability to close health disparities by assessing multilevel needs of persons with diabetes and connecting them to community resources [19]. Interventions delivered by community health workers increase their sociocultural acceptability [20]. DSMES interventions integrating community health workers have shown success in improving diabetes self-management and health in rural minority populations [21,22]. However, community health workers face obstacles when working in rural communities, such as transportation, limited resources, and limited supervision and support, which affect their work productivity and service quality [23,24]. Thus, providing sufficient support to community health workers is vital for successful community health worker programs in rural communities.

Integrating mobile health (mHealth) technology is a promising approach to improve DSMES access in nonclinical settings and to improve health services provided by community health workers [24,25]. Health care services included in the mHealth category rely on handheld mobile devices, including cell phones, tablets, and wearables, that enable mobile apps [26]. Evidence shows that the use of mHealth by community health workers improves communication, avoids unnecessary transportation, improves access to care resources, and results in positive effects on patient health [27,28]. However, the feasibility of a community health worker-led, mHealth-based DSMES program has not been evaluated in rural Latino persons with diabetes.

In this paper, we report results of a feasibility study to evaluate retention, delivery, usage, and acceptability of an mHealth-based DSMES program led by trained community health workers for Latino persons with diabetes living in rural South Texas. The impact of the intervention on weight loss and glycemic control was also explored.

## Methods

### Study Design and Recruitment

This was a single-arm, pre-post, National Institutes of Health Stage 1B study to examine the feasibility of combining an mHealth-based intervention that relied on community health worker facilitation to improve access to DSMES in a resource-poor rural community [22]. A 12-week intervention was delivered by 3 trained community health workers to 15 adults with type 2 diabetes living in rural South Texas. All community health workers were affiliated with the South Coastal Area Health Education Center, a local community health care intermediary aiming to improve access to quality health care for medically underserved communities in South Texas.

Participants were recruited through flyers posted at the Community Action Corporation of South Texas. Community health workers telephoned individuals who expressed interest in participation to assess their eligibility for enrollment. The inclusion criteria were as follows: (1) age ≥ 18 years, (2) a diagnosis of type 2 diabetes, (3) ability to read and write in English, (4) residency in Jim Wells County, (5) possession of a compatible smartphone with a data plan, and (6) readiness to make a lifestyle change. Participants were excluded if they were (1) on insulin treatment, (2) had a history of severe psychiatric disorder, (3) had difficulty in performing daily or regular activity, (4) had substance abuse issues, or (5) were planning a pregnancy or planning to breastfeed within the following 6 months. Participants gave verbal consent to participate. Prior to implementation of any study procedures, this project was reviewed by Office of the Institutional Review Board at the University of Texas Health Science Center at San Antonio and determined to be non-regulated research (HSC20190486N).

### Theoretical Framework

**Figure 1:**
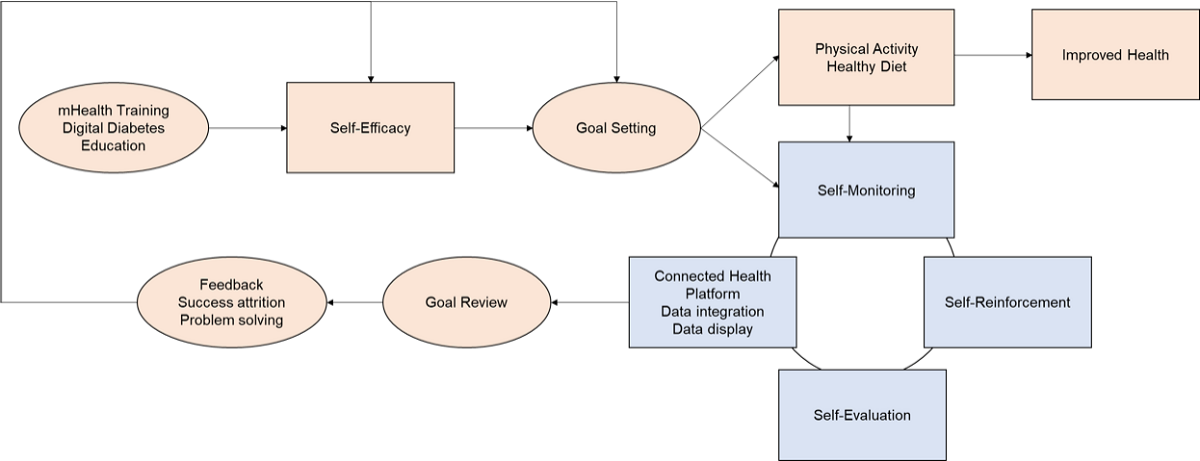
The “high-tech, high-touch” intervention model. Self-regulation theory and social cognitive theory provide theoretical support for the model. Self-regulation theory posits that self-monitoring aids self-evaluation of progress made towards one’s goals and self-reinforcement of progress (shown in blue). According to social cognitive theory, providing self-management knowledge and skills and support improves health behaviors by enhancing self-efficacy toward performing self-management behaviors (shown in orange). Community health workers are involved in the "high-tech, high-touch" model to provide diabetes self-management education and support services (shown by oval).

**Figure 2:**
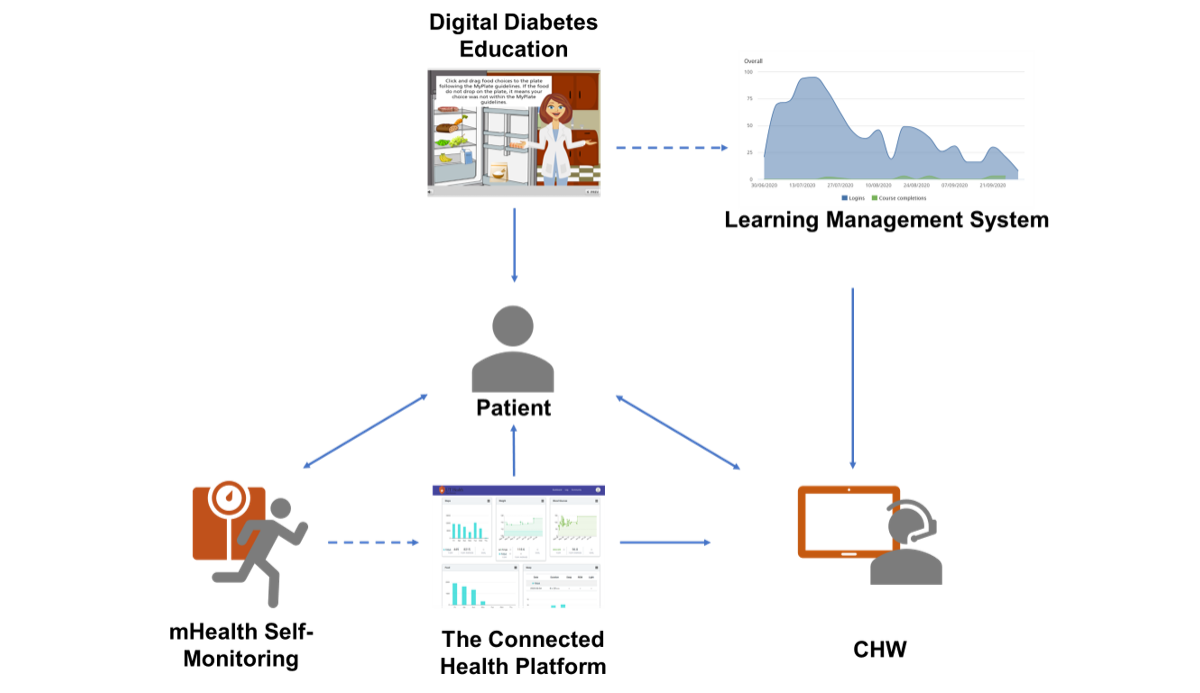
Intervention delivery flow diagram. Participants received digital diabetes education, performed mHealth-based self-monitoring, and maintained 2-way communication with the community health workers. Their performance was captured by the Connected Health Platform and the TalentLMS learning management system, which helped community health workers to provide personalized diabetes self-management educationand support services. CHW: community health worker.

Supporting behavior change is a key objective for DSMES programs [17]. This study was built upon self-regulation and social cognitive theory (SCT) [29] (Figure 1). Self-regulation theory posits that self-monitoring aids self-evaluation of progress made toward one’s goals and aids self-reinforcement of one’s progress. According to SCT, receiving self-management knowledge and skill support improves health behaviors by enhancing one’s self-efficacy and ability to perform the self-management behaviors. The development of the mHealth intervention was guided by the Behavior Information Technology model that links targeted diabetes self-management behaviors with evidence-based behavior change techniques underlying the theoretical mechanisms of SCT to address the goals of DSMES (see Figure 2) [30]. Table 1 explains the operationalizations of the behavior change techniques for the targeted diabetes self-management behaviors (ie, the mHealth tool and frequency of access or use by the study participants).

This intervention adopted a unique “high-tech, high-touch” approach. We integrated self-monitoring data from multiple mHealth tools into a cloud-based platform so that (1) participants could increase self-efficacy for behavior change to improve health outcomes by reviewing data on the platform, (2) community health workers could access the platform and address participant barriers remotely, thereby promoting participant self-efficacy for behavior change and diabetes self-management, and (3) community health workers could obtain support from the research team for complex diabetes self-management cases and technological issues via video conferencing.

**Table 1:** Use of behavior change techniques and mHealth tools.

Behavior change techniques	Operationalization	mHealth tools	Frequency
Instruction on how to perform a behavior	Advise the participant how to adhere to diet and exercise self-monitoring goals.	Digital diabetes education session	Weekly
Self-monitoring of behavior	Ask the participant to wear a fitness tracker; ask the participant to record food intake.	Electronic logs; passive data collection	Daily
Establish a method for the person to monitor and record the outcomes of their behavior	Ask the participant to weigh themselves; ask the participant to monitor blood glucose.	Passive data collection	Daily
Feedback on outcomes of behavior	Inform the participant of how much weight they have lost and their blood glucose level.	Phone call or videoconference	Weekly
Feedback on behavior	Inform participant of how they performed on diet and physical activity goals.	Phone call or videoconference	Weekly
Discrepancy between current behavior and goals	Point out out-of-range blood glucose readings. Point out that recorded diet and exercise fell short of the goal. Point out lack of adherence to self-monitoring goals.	Phone call or videoconference	Weekly

### Description of the Intervention

The pilot DSMES intervention was delivered over a 12-week period by community health workers via mHealth. Once enrolled, participants received mHealth and one-on-one training from community health workers (Table 2). To facilitate establishing rapport and communication, each community health worker was paired with 5 participants to provide individualized DSMES services.

To ensure fidelity of intervention delivery, all community health workers received a 1-day training session provided by a research staff member. The training covered a study description, diabetes self-management, data collection, and intervention delivery. During the intervention, the study team met with community health workers weekly to resolve problems they encountered, following the ECHO (Extension for Community Health Care Outcomes) model [31].

Weekly digital diabetes education was delivered to increase the participants’ diabetes knowledge and skills. Building upon the Diabetes Prevention Program Group Lifestyle Balance Program, the curriculum was tailored to local needs. For example, we modified group-based activities to add individual-based aligned lessons with the AADE7 (American Association of Diabetes Educators-7) framework [32]; tailored the content to the local culture; added interactive components, including videos, quizzes, and webpages; and reduced the content length to less than 10 minutes per lesson. All lessons were reviewed by community health workers. The lessons were developed using eLearning authoring software (Articulate Storyline; Articulate Global LLC).

Collaborative goal setting between the community health workers and participants with diabetes was integral for DSMES [32]. Each week, the participants set daily SMART (specific, measurable, achievable, relevant, and time-bound) goals pertaining to physical activity and diet. The community health workers assisted the participants in choosing goals for physical activity and diet. Additionally, the participants set self-monitoring goals for frequency of weight self-monitoring, food logging, physical activity tracking, and blood glucose self-monitoring in week 1. Participants met with community health workers on weeks 2, 4, 6, and 10 to review goal achievement, address barriers, and make necessary modifications.

**Table 2:** Devices and apps used for mHealth and their functions.

mHealth devices and apps	Functions
Fitbit Inspire fitness tracker (Fitbit LLC)	Physical activity goal setting and self-monitoring
Fitbit app (Fitbit LLC)	Dietary goal setting and food logging
Fitbit Air body scale (Fitbit LLC)	Weight loss goal setting and self-monitoring
BioTel Care glucose meter (BioTelemetry, Inc)	Blood glucose self-monitoring
TalentLMS (Epignosis LLC)	eLearning management system for delivering asynchronous diabetes education lessons
The Connected Health Platform	For community health workers to monitor participants’ progress toward goal achievement and provide ongoing support.
Online interactive diabetes education lessons created using Articulate Storyline 360 (Articulate Global LLC)	Education content for teaching diabetes self-management education and support services delivered to the study participants via TalentLMS.

### Connected Health Platform

**Figure 3:**
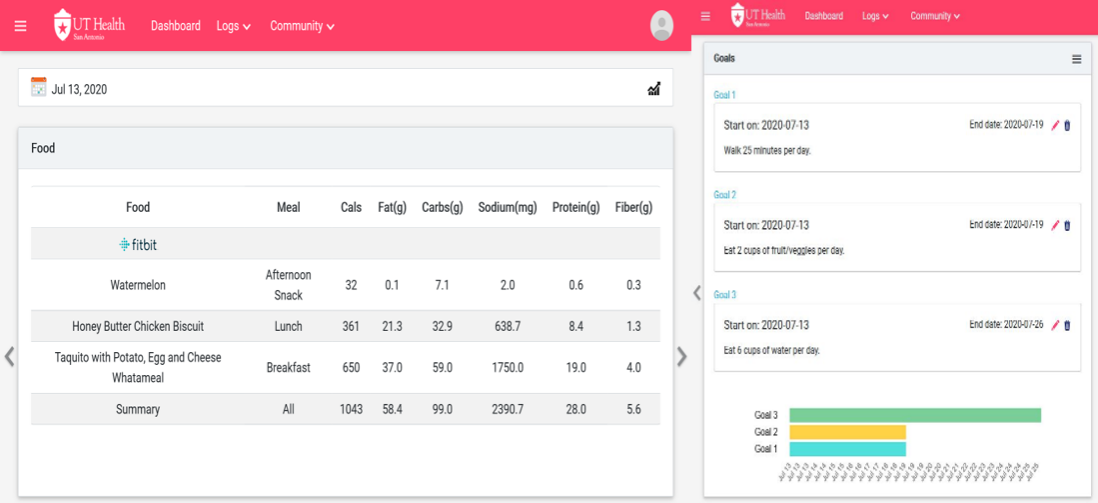
Screenshot of the Connected Health Platform. The Connected Health Platform displayed details about the diet of the participants with diabetes, including food type, macronutrient consumption, and calorie intake (left). The community health workers set nutrition and physical activity goals in the Connected Health Platform (right).

Data collected from the mHealth devices and apps was automatically synchronized and stored by the Connected Health Platform (Figure 3), an application programming interface integration platform developed by the study team [33]. This platform was designed to present self-monitoring data relevant to behavioral goals, as studies have found that combining physical activity and diet data with blood glucose self-monitoring has the potential to help community health workers provide personalized DSMES service [34].

Once logged, the community health workers could see 7-day plots of diet, including macronutrient details, calorie intake, and water consumption; activity, including exercise type, steps, sleep, and weight; blood glucose; and weekly behavioral goals. The community health workers could also select a date range to view self-monitoring data trends and interactions by overlaying multiple sources, and they could track participants’ progress on goals, thereby enabling problem-solving strategies during behavioral follow-up sessions. The participants were also allowed access to the platform. Additionally, the platform served as a data storage tool from which the research team could download data from various mHealth sources.

### Study Measures

We addressed feasibility according to retention, actual intervention use, barriers to implementation, satisfaction, and preliminary health effects.

#### Retention

Retention rate was the percentage of enrolled participants who completed posttest data collection.

#### Actual Intervention Use

Actual use of digital education was quantified as the weekly number of days the lessons were accessed. Actual use of self-monitoring devices was operationalized as the weekly number of days with self-monitored weigh-ins, food logs, step counts, and blood glucose readings. All community health workers kept a log to record their contact with participants; data included call duration and issues addressed. We operationalized use of behavioral change strategies as the cumulative number of behavioral goals set during the study and reported the percentage of time during the week the participants accomplished these goals.

#### Barriers to Implementation

The participants reported barriers to achieving behavioral goals during behavioral follow-up sessions; at posttest, they listed life events that affected diabetes self-care using the Recent Life Event Questionnaire [35].

#### Satisfaction

Satisfaction was measured using Customer Satisfaction Questionnaire short version (CSQ-8) [36]. The CSQ-8 is a 4-point Likert scale with 8 items. The total score ranged between 8 and 32, with a higher score indicating a higher program satisfaction rate. To measure the participants’ level of acceptance, we adapted the poststudy surveys used by Yin et al [37]. Specifically, the participants rated their level of satisfaction with each intervention component, its perceived benefits, and their confidence in continuing diabetes self-management. Patient satisfaction with digital education was measured at the end of each module on a 4-point Likert scale, with 1 indicating not acceptable; 2, fair acceptability; 3, good acceptability; and 4, excellent acceptability.

#### Preliminary Health Effects

All participants checked HbA_1c_ with their doctor pre- and posttest. Weight was self-measured to 0.1 lb (0.045 kg) and represented as the mean of 2 measurements. To assess participants’ responses to the intervention, we plotted weekly changes in average daily steps and self-measured body weight.

We also measured changes in eHealth literacy and outcome expectations. The 14-item eHealth Literacy Toolkit assesses eHealth literacy as technology confidence, attitudes toward engaging with technology, and mobile technology familiarity [38]. Items were answered on a 10-point scale, from “completely disagree” to “completely agree.” The 13-item Perceived Therapeutic Efficacy Scale (PTES) was used to assess perceived beliefs for the effect of each mHealth component on diabetes self-management [39]. Participants answered items on a 10-point scale (with 0 indicating “no confidence,” and 10 indicating “highest confidence”) [39].

#### Other Measures

At baseline, participants completed surveys on demographic information, including age, sex, educational background, language spoken, marital status, diabetes history, medication history, and experience with mHealth.

### Data Analysis

All descriptive statistics are reported as the mean (SD) for continuous variables and frequencies and relative frequencies for categorical variables. A 2-tailed paired *t* test was used to determine intervention effects on weight loss, HbA_1c_, technology efficacy, and PTES score. All statistical procedures were performed using R software (R Foundation for Statistical Computing). The threshold for statistical significance was a 2-sided *P* value of .05.

## Results

All 15 participants completed the baseline assessment; 1 participant had missing data for posttest HbA_1c_ (for a retention rate of 93%). All participants attended the behavioral follow-up sessions at weeks 1, 2, 4, 6, and 10. Detailed demographic characteristics of the study participants are presented in Table 3. Participants were all Latino and were mainly older adults, female, and had at least a high school education. All participants had at least one type of health insurance. Participants had a long history of diabetes diagnosis (mean 15.2, SD 11.9 years). Thirteen participants took at least one type of diabetes medication. Most participants had no experience with mHealth.

**Table 3:** Participant characteristics at baseline (N=15).

Characteristics		Value
Age (years), mean (SD)		61.87 (10.67)
Sex (female), n (%)		9 (60)
Preferred language (English), n (%)		12 (80)
Education (≥ high school), n (%)		14 (93)
Marital status (married or living with partner), n (%)		8 (53)
Living with someone (yes), n (%)		14 (93)
Insurance status (insured), n (%)		15 (100)
Current internet service needs met (yes), n (%)		15 (100)
**Answer to “How do you usually use the internet to search for health information?” n (%)**		
	Mobile phone	13 (87)
	Laptop or personal computer	7 (47)
	Tablet	6 (40)
	Work computer	2 (13)
	Public computer	1 (7)
	Other	1 (7)
**Answer to “How often do you use mobile health apps of any type?” n (%)**		
	Every day	1 (7)
	Several days a week	2 (13)
	About one day a week	0 (0)
	(Almost) never	12 (80)
**Answer to “How often do you use a digital health device?” n (%)**		
	Every day	4 (27)
	Several days a week	1 (7)
	About one day a week	1 (7)
	(Almost) never	8 (53)

### Actual Intervention Use

**Figure 4:**
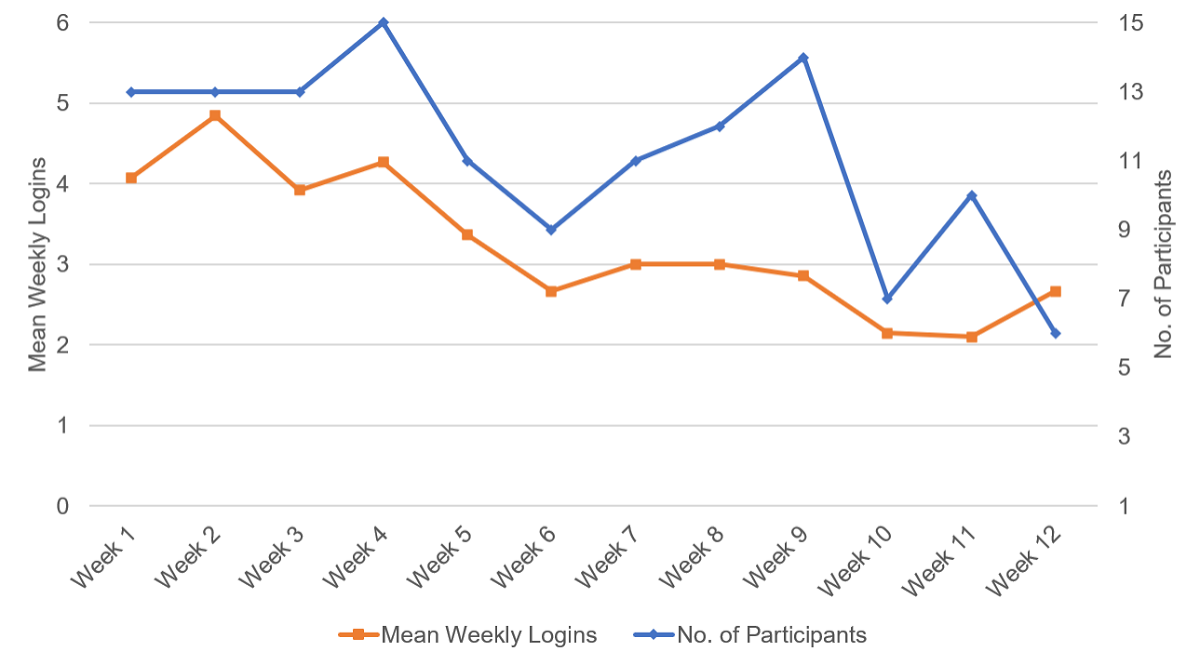
Mean weekly digital lesson logins and mean number of participants accessing digital lessons by week of the trial, showing the mean weekly logins (in orange) and the number of participants accessing the digital lessons per week (in blue) over the 12-week study period. Mean weekly logins represents the number of times participants logged into the digital lessons. The number of participants is the number of participants that used the app at least once in that week.

Digital education completion rates were high (mean 87.5%, SD 22.5%) with most (14/15, 93%) participants accessing all digital education modules and 8/15 (53%) completing all lessons. The participants accessed digital education modules multiple times during the week (2-5 times). There was a continual decline in the number of active users and average weekly logins as the study proceeded (Figure 4). The highest weekly logins occurred in week 4, with the 15 participants logging in an average of 4.3 times.

**Figure 5:**
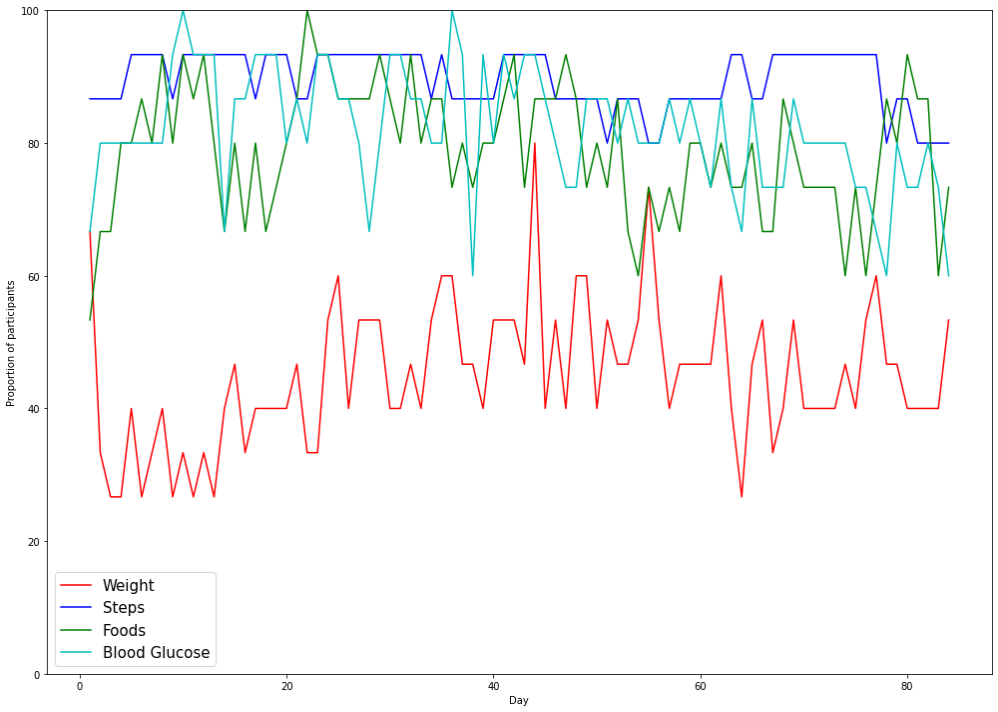
Proportion of participants using the mHealth self-monitoring technologies by day of the trial.

During the 12-week (84-day) intervention, the mean percentage of days that the participants self-monitored steps, food, blood glucose, and weight were 89% (SD 21%), 78% (SD 21%), 47% (SD 13%), and 81% (SD 16%), respectively. Overall, the percentage of participants who used self-monitoring technologies daily did not change significantly during the study course (Figure 5).

All participants set daily physical activity and diet goals with the community health workers and performed well in accomplishing these goals, as reported by the Connected Health Platform. Nearly one-third of the participants met their diet and physical activity goals on all days of the week. Most participants accomplished their daily dietary goals on more than half of the days of the week (Table 4).

On average, each participant made 16.73 (SD 8.0) calls to their community health worker, with each call lasting between 5 minutes and 2.5 hours.

**Table 4:** Level of behavioral goal achievement captured by the Connected Health Platform. The total number of dietary goals was 172; the total number of physical activity goals was 93.

Reported percentage of time during the week participants achieved goals	No time (0%)	Little of the time (25%)	Some of the time (50%)	Most of the time (75%)	All the time (100%)
Dietary goals, n (%)	5 (3)	11 (6)	41 (24)	68 (40)	47 (27)
Physical goals, n (%)	15 (16)	17 (18)	10 (11)	18 (19)	33 (36)

### Barriers to Implementation

Participants reported different types of barriers to completing the intervention activities. The most reported barriers were “motivation” for food logging, “time” for exercise self-monitoring, “technical” for weight self-monitoring, and “forgetfulness” for blood glucose self-monitoring.

The COVID-19 pandemic appeared to affect diabetes control. Participants reported the following life events that affected diabetes control: “having relatives or close friends seriously ill, injured, or die” (7/15, 47%), “having immediate family be seriously ill, injured, or die” (4/15, 27%), and “having major financial difficulties” (3/15, 20%).

### Satisfaction

Overall, the participants were satisfied with the intervention (Table 5). The mean score for CSQ-8 was 29.53 (SD 3.04). All participants agreed that the community health workers provided needed support, and they liked the support. Participants agreed that the program helped them be active and eat healthfully. Participants also expressed the intention to continue the intervention activities. Most of the participants (14/15, 93%) indicated confidence to continue blood glucose self-monitoring and healthful eating (Table 5) and blood glucose self-monitoring and healthful eating became a high or essential priority for most participants.

The participants rated most digital education modules as “good.” The highest score was given to the “Healthy Eating” lesson and the lowest score was given to the lesson on “Motivation” (Table 6).

**Table 5:** Poststudy survey on intervention satisfaction (N=15).

Questions	Strongly agree, n (%)	Agree, n (%)	Disagree/strongly disagree, n (%)
Did the program help you to be more physically active?	8 (53)	7 (47)	0 (0)
Are you still being active with the information from the program?	6 (40)	7 (47)	2 (13)
Did the program help you to eat healthy?	6 (40)	9 (60)	0 (0)
Are you still eating healthy with the information from the program?	6 (40)	8 (53)	1 (7)
Did the program help you to lose weight?	6 (40)	7 (47)	2 (13)
Did the program help you control you blood glucose?	5 (33)	8 (53)	2 (13)
Are you still trying to lose weight with the information from the program?	6 (40)	8 (53)	1 (7)
I liked the digital diabetes education lessons.	7 (47)	7 (47)	1 (7)
I learned how to change my lifestyle with information from the health education lessons.	7 (47)	6 (40)	2 (13)
Information in the health lessons was easy to understand.	9 (60)	5 (33)	1 (7)
I liked the support calls and text messages from the community health worker.	10 (67)	5 (33)	0 (0)
The community health worker provided the support I needed.	12 (80)	3 (20)	0 (0)
I liked setting the health goals.	5 (33)	9 (60)	1 (7)
I liked the videos on physical activity and diet.	5 (33)	8 (53)	2 (13)
I liked the videos with information on obesity and diabetes.	5 (33)	8 (53)	2 (13)
Compared with when the program started in the summer, I am confident that I can continue to exercise regularly now.	5 (33)	4 (27)	2 (13)
Compared with when the program started in the summer, I am confident that I can continue eating healthy now.	6 (40)	8 (53)	0 (0)
Compared with when the program started in the summer, I am confident that I can continue to monitor my blood sugar now.	10 (67)	5 (33)	0 (0)

**Table 6:** Digital diabetes education topics, session completion, session evaluation, and lesson completion rate.

Session topics and lessons		Rating (mean)^a^	Completion Rate (%)
**1. Diabetes self-management**		3.07	87
	Diabetes self-management activities		93
	Diabetes self-management skills		100
	Be an active self-manager		60
	Problem solving		93
**2. Managing and monitoring your behavior**		3.00	92
	Blood glucose self-monitoring		60
	Monitor your diet and weight		100
	New ways to tip the calorie balance		100
	Portion control		93
	Food and nutrition labels		100
	Monitor your exercise and physical activity		100
	Exercise caution		87
	Use digital tools to support your diabetes management		93
	Quick tips to maintain a healthy lifestyle		93
**3. Healthy plate**		3.21	83
	MyPlate: planning a meal		53
	Start simple with MyPlate webtool		73
	Resource: MyPlate action guide		100
	Resource: MyPlate message toolkit		87
	Learn to create your smart goal		93
**4. Planning: healthy rating and physical activity**		3.07	91
	Community facilities		93
	Healthy eating and food preparation		87
	Exercise videos		93
**5. Maintaining overall health**		3.00	83
	Be mindful: eating, exercise, and stress management		87
	Maintain behavioral goals and gain social support		80
**6. Taking medication**		3.00	60
	Diabetes medication and provider communication		60
**7. Eating healthy away from home**		3.21	87
	Healthy eating on a budget		80
	Healthy shopping tour		87
	Healthy dining out		87
	Problem and helpful social cues		93
**8. Motivation techniques: how to stay motivated**		2.86	85
	How to stay motivated		80
	Be good to yourself		87
	What is your purpose now		87
	Social support		87

^a^Participants rated the content of the lessons on a 4-point Likert scale at the end of each session (1, not acceptable; 2, fair; 3, good; and 4, excellent).

### Preliminary Effects

**Figure 6:**
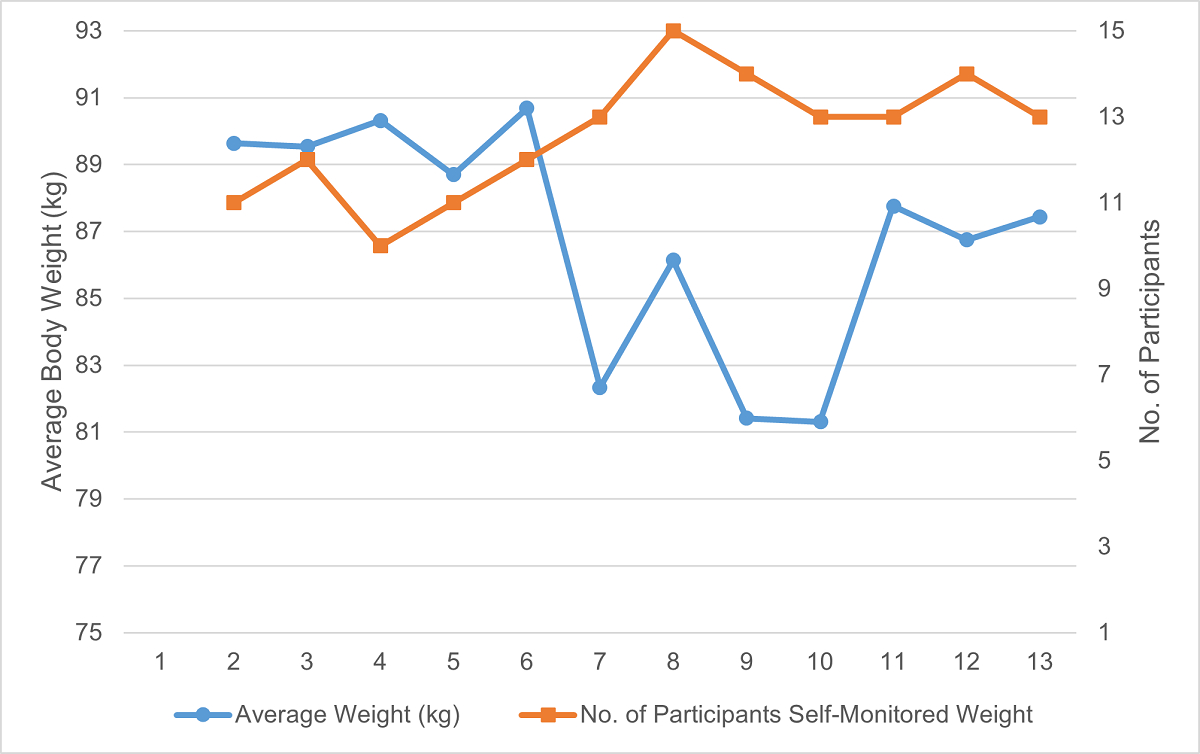
Average body weight (in kg) by week.

**Figure 7:**
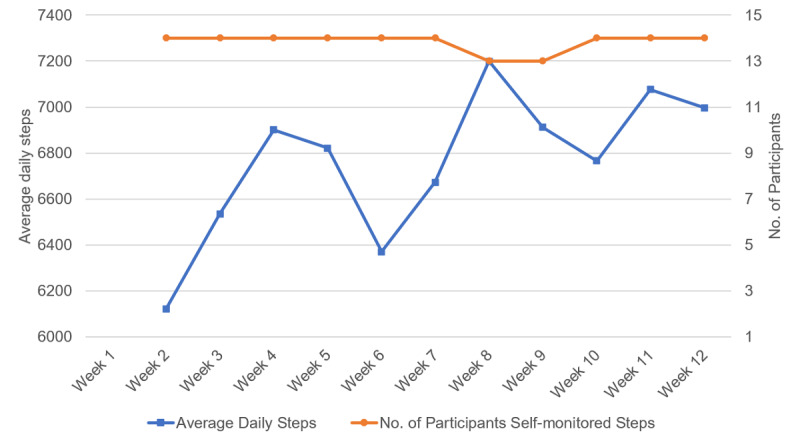
Average daily steps by week.

Participants showed a significant reduction in body weight of 3.5 (SD 3.2) kg (*P*=.001) from baseline to posttest (Table 7). HbA_1c_ did not change significantly. We observed weekly improvement in weight loss and steps (Figure 6, Figure 7). Participants achieved the largest weight loss between weeks 6 and 10, when the greatest improvement in average steps was also observed.

**Figure 8:**
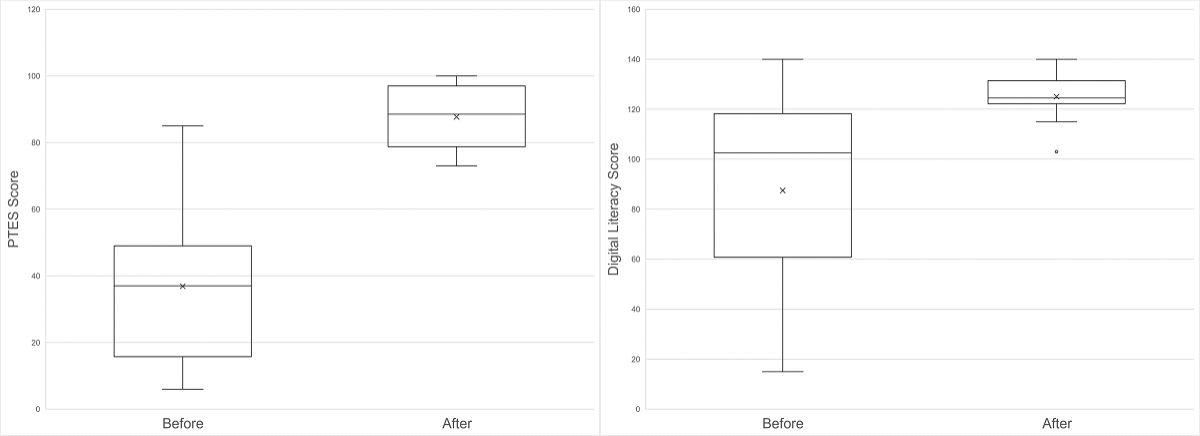
Pre- to poststudy changes in perceived therapeutic efficacy scale score (left) and eHealth literacy score (right). PTES: Perceived Therapeutic Efficacy Scale.

Participants showed a significant improvement in PTES score of 50.86 (95% CI 36.61-65.10; *t*_13_=7.71; *P*<.001) and eHealth literacy by 37.57 from baseline to posttest, (95% CI 16.72-58.42; *t*_13_=3.89; *P*<.001) (Figure 8).

**Table 7:** Changes in preliminary efficacy outcomes from baseline to 3 months (N=14).

Outcomes	Baseline mean (SD)	3-month posttest mean (SD)	*t* test (*df*) (pre-post)	*P* value
Weight (kg)	86.1 (25.9)	82.6 (24.1)	1.9 (14)	.001
HbA_1c_ (%)	6.91 (1.28)	7.04 (1.66)	–.44 (13)	.668

## Discussion

### Principal Findings

Latinos living in rural South Texas suffer high rates of diabetes, but their access to DSMES is poor. To address multidimensional access barriers, we designed an innovative DSMES intervention, combining the community health worker model and mHealth technologies. To our knowledge, this is the first published study to examine the combined impact of community health worker facilitation and mHealth in improving access to DSMES in a rural Latino population. Our findings demonstrate that an mHealth-based, community health worker-led approach is a feasible and acceptable means to augment DSMES services in South Texas. We found that mHealth facilitated community health worker engagement in delivering DSMES services. The intervention was well-received by the participants, as evidenced by their consistent use of mHealth and frequent participant-community health worker interaction. The participants reported a high level of satisfaction with the intervention. Moreover, we observed improvements in self-care behaviors and health outcomes. Taken together, this study suggests that our unique “high-tech, high-touch” solution has the potential to be tested for efficacy in larger randomized controlled trials.

Our findings show that by using mHealth tools, community health workers can address the multidomain health determinants proposed by the National Institute on Minority Health and Health Disparities framework [16]. For example, we addressed individual knowledge and skill gaps, helped persons with diabetes overcome literacy barriers for using mHealth (individual-behavioral domains), and offered culturally tailored digital diabetes education (sociocultural environment) [20].

We achieved a higher retention rate than previous diabetes self-management programs conducted in rural communities [40,41] and observed consistent mHealth usage. High dropout rates and a decline in attendance are commonly reported in DSMES mHealth interventions [42]. While equipping participants with various technologies can overcome geographical and temporal barriers, the participation of community health workers allowed frequent communication with persons with diabetes and the maintenance of their participation. Previous rural DSMES interventions found a positive relationship between community health worker-participant telephone contacts and attendance rate [6]. Therefore, our findings demonstrate the value of adding community health workers to a high-tech program and show that this approach facilitates ongoing participation.

Consistent with other rural-based DSMES interventions, our sample reported high rates of satisfaction [6]. We speculate that such high satisfaction is due to a strong desire to feel supported and “cared for” among the population [43]. Participants’ frequent use of the intervention strengthened this speculation. Therefore, a “high-tech, high-touch” intervention may address diabetes care disparities in South Texas and other underserved areas with similar features.

Low health and eHealth literacy are major barriers to implementing mHealth in underserved communities [43]. Our participants had little to no prior experience with mHealth. They also reported low expectations for mHealth on diabetes control at baseline. These low expectations did not limit mHealth engagement, as most participants started using mHealth in week 1 and used it consistently throughout the project period. In addition, the participants seldom reported technology as a barrier to diabetes self-care. The participants showed significant improvements in mHealth outcome expectancy and eHealth literacy at 12 weeks. Our findings indicate that a “high-touch, high-support” model might help persons with diabetes overcome health and eHealth literacy barriers and allow them to use mHealth for diabetes self-care. Looking forward, we will test this model with a longer follow-up period to determine if support from community health workers is sufficient for participants with diabetes to maintain diabetes self-care over the long term.

The high level of intervention acceptability could also be attributed to our integration of multiple personalization strategies [44]. Considering the complex needs of persons with diabetes, we acknowledge the irreplaceable role of community health workers in dynamic personalization strategies. Specifically, the community health workers were able to tailor DSMES to cultural values and context, literacy and numeracy abilities, and personal beliefs and concerns in real time, with mHealth as a complement to the personalization strategies [43]. For example, integrating self-monitoring data into the Connected Health Platform made it easier for the community health workers to provide personalized feedback [34]. To continuously engage persons with diabetes in DSMES, future research needs to leverage multidimensional personalization strategies enabled by community health workers and mHealth.

Poor integration of technology into health professionals’ workflow could increase their workload and discourage them from adopting mHealth interventions. Considering that community health workers are a valuable resource that can affect intervention scalability [45], we aimed to integrate mHealth into community health workers’ workflow with minimal barriers. Presenting a large amount of patient-generated self-monitoring data in an informative format is important for reducing the burden placed on community health workers by mHealth interventions [46]. We learned from a previous study that diabetes educators prefer a centralized system that allows a flexible view of self-monitoring information from persons with diabetes [34]. Therefore, we used the Connected Health Platform to allow community health workers to merge their preferred self-monitoring data and observe interactions in diet and activity with blood sugar or weight, which enabled them to quickly evaluate the self-care progress of participants with diabetes. Community health workers also met weekly with the study team via video conference, which may have raised their motivation and performance, resulting in improved DSMES service quality [47].

Consistent with previous DSMES programs in underserved communities, participants achieved 4.1% weight loss at 3 months. Participants also maintained good glycemic control at 3 months, similar to previous short-term mHealth-based DSMES interventions [35,36]. While a meta-analysis reported a 0.4% HbA_1c_ reduction after mHealth interventions in persons with diabetes, these studies were conducted in persons with diabetes with poor baseline HbA_1c_ and had longer study durations [37]. These findings warrant future examination of our intervention in rural Latinos with poorer glycemic control and extension of the study duration to examine the long-term effects of the intervention.

This study was conducted during the COVID-19 pandemic. At the time of the study, Latinos represented 43.5% of confirmed COVID-19 deaths in Texas [48]. Moreover, persons with diabetes experienced greater challenges during the pandemic, including more severe symptoms, a higher mortality rate, limited health care resources, concerns related to cross-infection, and emotional stress. [49]. Therefore, our study provides a timely digital solution to address the unique needs of persons with diabetes and to optimize allocation of health care resources in underserved communities.

### Limitations

Several limitations need to be considered in interpreting our study findings. First, the duration was shorter than typical DSMES programs. Given that diabetes self-management requires long-term commitment, our study needs to be extended with a longer follow-up period. Second, the statistical findings of our study need to be interpreted with caution due to the small sample size. Third, we only included participants who had a digital data plan, which could limit the generalizability of our findings to rural Latinos with diabetes. Fourth, we only measured actual use of the intervention by the participants. Future studies should consider collecting data from community health workers to complement data from persons with diabetes to inform intervention scalability. Lastly, we did not collect data on recruitment, which could have provided valuable information on the potential reach of our program [50].

### Conclusions

Our findings suggest that a “high-tech, high-touch” approach holds promise to reduce DSMES access barriers faced by rural Latino residents of South Texas. We found that mHealth facilitated self-care and remote monitoring by participants with diabetes, interaction between community health workers and participants with diabetes, and enabled community health workers to actively contribute to providing DSMES services. In the future, our findings should be tested in a fully powered randomized controlled trial to examine the efficacy of our methods in improving access to quality DSMES and glycemic control in persons with diabetes living in resource-poor rural communities.

## Abbreviations

CSQ-8: Customer Satisfaction Questionnaire short version
DSMES: Diabetes self-management education and support
HbA_1c_: Hemoglobin A_1c_
mHealth: Mobile Health
PTES: Perceived Therapeutic Efficacy Scale
SCT: social cognitive theory
